# Expression of Genes Related to Phenylpropanoid Biosynthesis in Different Organs of *Ixeris dentata* var. *albiflora*

**DOI:** 10.3390/molecules22060901

**Published:** 2017-05-30

**Authors:** Sang-Hoon Lee, Yun-Ji Park, Sang Un Park, Sang-Won Lee, Seong-Cheol Kim, Chan-Sik Jung, Jae-Ki Jang, Yoonkang Hur, Yeon Bok Kim

**Affiliations:** 1 Department of Herb Crop Resources, National Institute of Horticultural & Herbal Science, RDA, Eumseong-gun 27709, Korea; omega119@naver.com (S.-H.L.); swlee1004@korea.kr (S.-W.L.); kimsec@korea.kr (S.-C.K.); jung100@korea.kr (C.-S.J.); changjk@korea.kr (J.-K.J.); 2 Department of Biology, Chungnam National University, 99 Daehak-ro, Yuseong-gu, Daejeon 34134, Korea; ykhur@cnu.ac.kr; 3 Department of Crop Science, Chungnam National University, 99 Daehak-ro, Yuseong-gu, Daejeon 34134, Korea; yunji0825@hanmail.net (Y.-J.P.); supark@cnu.ac.kr (S.U.P.)

**Keywords:** *Ixeris dentata* var. *albiflora*, phenylpropanoid, transcript level

## Abstract

Members of the genus *Ixeris* have long been used in traditional medicines as stomachics, sedatives, and diuretics. Phenylalanine ammonia-lyase (PAL), cinnamate-4-hydroxylase (C4H), 4-coumarate: coenzyme-A (CoA) ligase (4CL), chalcone synthase (CHS), and dihydroflavonol 4-reductase (DFR) are important enzymes in the phenylpropanoid pathway. In this study, we analyzed seven genes from *Ixeris dentata* var. *albiflora* that are involved in phenylpropanoid biosynthesis, using an Illumina/Solexa HiSeq 2000 platform. The amino acid sequence alignments for *IdPALs*, *IdC4H*, *Id4CLs*, *IdCHS*, and *IdDFR* showed high identity to sequences from other plants. We also investigated transcript levels using quantitative real-time PCR, and analyzed the accumulation of phenylpropanoids in different organs of *I. dentata* var. *albiflora* using high-performance liquid chromatography. The transcript levels of *IdC4H*, *Id4CL1*, *IdCHS*, and *IdDFR* were highest in the leaf. The catechin, chlorogenic acid, ferulic acid, and quercetin contents were also highest in the leaf. We suggest that expression of *IdC4H*, *Id4CL1*, *IdCHS*, and *IdDFR* is associated with the accumulation of phenylpropanoids. Our results may provide baseline information for elucidating the mechanism of phenylpropanoid biosynthesis in different organs of *I. dentata* var. *albiflora*.

## 1. Introduction

*Ixeris dentata* var. *albiflora*, a perennial herbaceous plant in the genus *Ixeris* (Asteraceae), is well known as an edible wild vegetable in Korea, China, Japan, and Mongolia. It has a pleasantly bitter flavor. Additionally, it has been used in Korea, China, and Japan for treatment of mithridatism, calculus disease, indigestion, pneumonia, hepatitis, and tumors [1]. Extensive research has been carried out to characterize the neuroprotective, anti-diabetic, and anti-allergic effects of polyphenol and phenolic compounds [2,3,4]. Phenolic compounds synthesized by various enzymes, as well as other secondary metabolites, are found in the edible parts of higher plants such as fruits, vegetables, cereals, and legumes [5]. Vrhovsek et al. [6] quantified 135 phenolics, such as benzoates, phenylpropanoids, coumarins, stilbenes, dihydrochalcones, and flavonoids in fruits. Some phenolic compounds are associated with defense against ultraviolet radiation and antioxidants [7,8]. In addition, they play a protective role through their antioxidant activities. These compounds are essential for the growth of plants and are also involved in plant reproduction. Plant-based foods have been reported to contain many phenolic compounds, which can be grouped into different classes according to their basic chemical structure [9]. Most of these compounds have received considerable attention as being potentially beneficial to health owing to their potent antioxidant activity as well as their hepatoprotective, hypoglycemic, and antiviral activities [9]. Recently, researchers from China reported that phenolic compounds from the rhizomes of *Smilax china* have anti-inflammatory activity [10]. Phenolic compounds in higher plants can be roughly divided into non-flavonoid and flavonoid compounds [11]. Flavonoids are the largest group of secondary metabolites in plants. They are further classified into flavonols, flavones, flavanones, catechins, anthocyanidins, and chalcones [12]. 

**Scheme 1:**
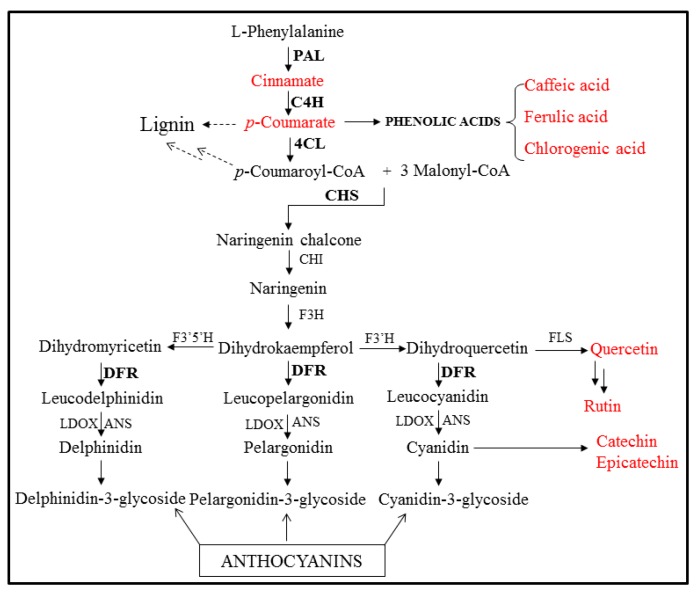
Phenylpropnaoid pathway in plants. PAL, phenylalanine ammonia-lyase; C4H, cinnamate 4-hydroxylase; 4CL, 4-coumaryl-CoA ligase; CHS, chalcone synthase; CHI, chalcone isomerase; F3H, flavone 3-hydroxylase; F3′H, flavonoid 3′-hydroxyase; F3′5′H, flavonoid 3′5′-hydroxyase; FLS, flavonol synthase; DFR, dihydroflavonol 4-reductase; ANS, anthocyanidin synthase; LDOX, Leucoanthocyanidin dioxygenase. The black bold and red letters indicate analyzed genes and compounds in this study, respectively.

Expressed sequence tag (EST) libraries have been generated for some medicinal plants, and the EST sequences have been catalogued in the dbEST database at the National Center for Biotechnology Information (NCBI). Many ESTs have been generated in an effort to identify genes involved in secondary metabolite biosynthesis and to develop molecular markers [13]. Next-generation sequencing (NGS) is a powerful and cost-effective tool for genome sequencing, miRNA expression profiling, genome re-sequencing, and DNA methylation analysis [14]. In recent years, de novo RNA transcriptome sequencing has been used for identification of functional genes in non-model organisms. To conduct a gene expression comparison of *I. dentata* tissues, we employed the RNA-Seq method using an Illumina Hi-Seq 2000 platform to generate a large quantity of transcriptome sequences from *I. dentata*. We isolated three genes that encode important enzymes in the phenylpropanoid pathway: phenylalanine ammonia-lyase (PAL), 4-coumarate: coenzyme-A (CoA) ligase (4CL), and chalcone synthase (CHS). Phenylpropanoid biosynthesis starts with the formation of the aromatic amino acid phenylalanine. PAL catalyzes the conversion of phenylalanine into cinnamic acid. Cinnamate 4-hydroxylase (C4H) and 4CL then catalyze the conversion of cinnamic acid to *p*-coumaroyl-CoA, which is the precursor for many phenylpropanoid products. Anthocyanins, flavonols, and isoflavonoids are synthesized from *p*-coumaroyl-CoA through a complex phenylpropanoid pathway [13] (Scheme 1).

Previously, our group has reported the transcript levels of flavonoid biosynthetic genes and the accumulation of phenolic compounds in common buckwheat, mulberry, radish, and Chinese cabbage [13,14,15,16]. However, analysis of gene expression and characterization of phenolic compounds in different organs of *I. dentata* have not been reported. In this study, we investigated the accumulation of phenolic compounds and the transcript levels of genes involved in the regulation of phenylpropanoid biosynthesis in different organs of *I. dentata*. 

## 2. Results and Discussion

### 2.1. Isolation and Sequence Analysis of Phenylpropanoid Biosynthetic Genes from I. dentata 

Seven genes (*IdPAL1*,*2*, *IdC4H*, *Id4CL1*,*2*, *IdCHS*, and *IdDFR*) related to phenylpropanoid biosynthesis were identified following next-generation sequencing of *I. dentata* (NGS, Illumina/Solexa HiSeq2000 platform, unpublished). The open reading frame (ORF) of IdPAL1 was 2136 bp and encoded a protein containing 711 amino acids, with a predicted theoretical molecular weight (MW) of 77.49 kDa and a deduced isoelectric point value (p*I*) of 5.89. The partial *IdC4H* had 1623 bp and 540 aa, with a predicted MW of 62.03 kDa and a deduced p*I* of 9.21. Similarly, the *IdPAL2* ORF had 2139 bp and 712 amino acids, with a predicted MW of 77.42 kDa and a deduced p*I* of 5.89. The ORFs of *Id4CL1* and partial *Id4CL2* were 1623 and 1749 bp, encoding 540 and 583 aa, respectively. The ORF of *IdCHS* and *IdDFR* had 1212, 1086 bp and 403, 361 aa, with a predicted MW of 44.0, 40.60 kDa and a deduced p*I* of 6.33, 5.67, respectively. The predicted MW of *Id4CL1* and *Id4CL2* were 59.04 and 63.29 kDa, respectively, and the deduced p*I* values were 5.36 and 6.36, respectively. The CHS ORF had 1212 bp and 403 amino acids, with a predicted MW of 44.0 kDa and a deduced p*I* of 6.33. From NCBI BLAST analysis of the deduced amino acid sequences, *IdPALs*, *IdC4H*, *Id4CLs*, *IdCHS*, and IdDFR showed high homology (between 82% and 96%) with genes from other plants. *IdPALs* contained a conserved motif (GTITASGDLVPLSYIA) between amino acid positions 209 and 224 (Supplemental Material Figure S1) in common with other plants [17]. *Id4CLs* were found to have a conserved motif (AMP binding domain, SSGTTGLPKGV; GEICIRG) (Supplemental Material Figure S2) [18,19]. Additionally, *IdCHS* had four CHS-specific conserved motifs (Supplemental Material Figure S3) [20].

### 2.2. Expression Profiles of IdPALs, IdC4H, Id4CLs, IdCHS, and IdDFR in Different Organs

**Figure 1:**
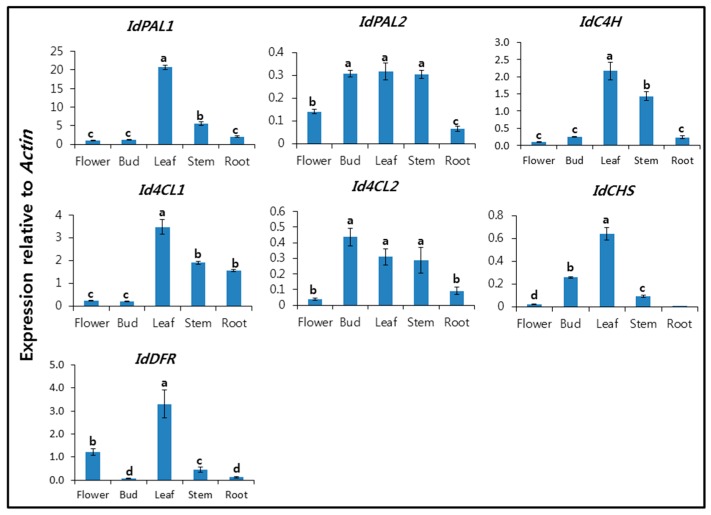
Expression levels of *IdPALs*, *IdC4H*, *Id4CLs*, *IdCHS*, and *IdDFR* in the flower, bud, leaf, stem, and root of *I. dentata*. Each value is the mean of three biological replicates, and error bars indicate standard deviation. Lower-case letters indicate statistically significant differences (*p* < 0.05) between different organs according to Duncan’s Multiple Range Test (*n* = 5).

The expression patterns of *IdPALs*, *IdC4H*, *Id4CLs*, *IdCHS*, and *IdDFR* were analyzed in the flower, bud, leaf, stem, and root using qRT-PCR (Figure 1). Our group previously investigated the expression patterns of phenylpropanoid biosynthetic genes in buckwheat [21], garlic [22], mulberry [14], and Chinese cabbage [16]. In the current study, we observed various expression patterns among phenylpropanoid biosynthetic gene isoforms in different organs. The expression levels of *IdPAL1*, *IdC4H*, *Id4CL1*, *IdCHS*, and *IdDFR* were the highest in the leaf, whereas *Id4CL2* expression levels were similar in the bud, leaf, and stem. *IdPAL2* and *Id4CL2* showed similar expression patterns. The transcript level of *IdPAL1* was approximately 4- to 20-fold higher in the leaf than in the flower, bud, stem, and root. *IdPAL1* showed high expression compared with *IdPAL2*. In *Scutellaria baicalensis*, three isoforms of PAL were isolated and investigated, showing different expression patterns in different organs. The transcript levels of *SbPAL1*, *SbPAL2*, and *SbPAL3* were highest in the stem, leaf, and root, respectively [23]. The transcript level of *IdC4H* was approximately 1.6- to 22-fold higher in the leaf than in the flower, bud, stem, and root. The expression pattern of *IdPAL1* was similar to that of *Id4CL1*. The transcript level of *Id4CL1* in the leaf was 14-fold higher than that in the flower and bud, whilst *Id4CL2* levels in the bud were 4.8 and 11.2 times higher than those in the root and flower, respectively. The transcript level of *Pv4CL1* was highest in the flower, whereas that of *Pv4CL2* was highest in the root [24]. In addition, it was reported that *Fe4CL1* exhibited highest expression in buckwheat roots, whereas *Fe4CL2* was most strongly expressed in stems [21]. Leaf expression of *IdCHS* was 2.5-, 6.9-, and 32.0-fold higher than that in the bud, stem, and flower, respectively, whereas it was rarely expressed in the root. *IdDFR* was 3.0-, 6.6-, and 33.0-fold higher than that in the flower, stem, and root, respectively. *IdPAL1* showed the highest expression level relative to *Actin*, compared with *IdPAL2*, *IdC4H*, *Id4CLs*, *IdCHS*, and *IdDFR.* Thus, the expression of *IdPAL1*, *IdC4H*, *Id4CL1*, *IdCHS*, and *IdDFR* was the highest in the leaf. To date, our group has investigated the expression patterns of phenylpropanoid biosynthetic genes in various plants [21,22,23,24,25,26]. Richet et al. [27] suggested phenylpropanoid gene expression in different organs of poplar could be altered by ozone exposure. In addition, methyl jasmonate, wounding, yeast extract, and silver nitrate treatments induced expression of phenylpropanoid biosynthetic genes in *S. baicalensis* and *Agastache rugosa* cell culture [23,28].

### 2.3. Analysis of Phenylpropanoids in Different Organs of I. dentata

**Figure 2:**
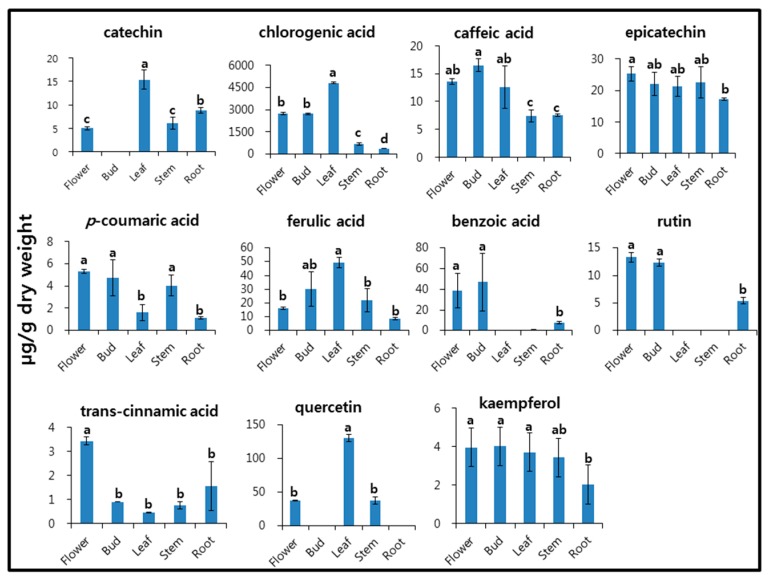
Accumulation of phenolic compounds in different organs of *I. dentata*. Each value is the mean of three biological replicates, and error bars indicate standard deviation. Lower-case letters indicate statistically significant differences (*p* < 0.05) between different organs according to Duncan’s Multiple Range Test (*n* = 5).

The phenylpropanoid compounds catechin, chlorogenic acid, caffeic acid, epicatechin, *p*-coumaric acid, ferulic acid, benzoic acid, rutin, trans-cinnamic acid, quercetin, and kaempferol—which have various health effects—were analyzed in the flower, bud, stem, leaf, and root of *I. dentata* (Figure 2). Catechin, chlorogenic acid, ferulic acid, and quercetin levels were highest in the leaf, whereas benzoic acid and rutin were not detected in the leaf. Additionally, *p*-coumaric acid, rutin, and trans-cinnamic acid were detected in the flower. The catechin content of the leaf (15.38 µg/g dry weight [DW]) was three-fold higher than that of the flower (5.00 µg/g DW), and catechin was not detected in the flower bud. It has been reported that catechin content is correlated with color pigment in buckwheat [29]. No significant differences in epicatechin levels were observed between any organs. The chlorogenic acid content of the leaf (4815.69 µg/g DW) was 7.4- and 13.3-fold higher than that of the stem (653.92 µg/g DW) and root (363.08 µg/g DW), respectively. Levels of benzoic acid were distributed in the following order: bud (46.88 µg/g DW) > flower (38.91 µg/g DW) > root (7.34 µg/g DW) > stem (0.65 µg/g DW). Rutin content was highest in the flower, and it was not detected in the stem or leaf. In *F. esculentum*, the highest rutin content was shown to be in the leaf, followed by the stem, seed, and root [21]. Rutin has a beneficial effect and is a healthy source of dietary fiber. Its content depends on the plant variety and environmental factors [30,31]. The quercetin content of the leaf was 3.5 times higher than that of the flower and stem. Kaempferol levels showed no significant differences across all organs. 

Phenylpropanoid or flavonoid metabolism has not previously been reported in *I. dentata*. This study is the first report on cloning and characterization of PALs, C4H, 4CLs, CHS, and DFR from *I. dentata*. However, in previous studies, gene expression related to rosmarinic acid (RA) biosynthetic pathways (PAL, C4H, 4CL, CHS, and CHI) in *A. rugosa* [32] and *P. vulgaris* has been examined [24]. The transcript levels of *IdPAL1*, *IdC4H*, *Id4CL1*, *IdCHS*, and *IdDFR* were the highest in the leaf, where catechin, chlorogenic acid, ferulic acid, and quercetin were also most abundant in *I. dentata*. Hence, we suggest that *IdPAL1*, *IdC4H*, *Id4CL1*, *IdCHS*, and *IdDFR* are related to the accumulation of catechin, chlorogenic acid, ferulic acid, and quercetin. It has been reported that *PvPAL*, *PvC4H*, and *Pv4CL1* are highly related to RA biosynthesis in *P. vulgaris* [24]. Additionally, various studies have shown a correlation between flavonoid accumulation and genes for *PAL*, *C4H*, and *4CL* in many species [33,34,35,36]. In addition, gene expression of *AgPAL* and *AgC4H* has been reported to be related to the production of secondary metabolites in *A. gigas* [36]. Based on these results, we speculate that trans-cinnamic acid, rutin, and epicatechin content was highest in the flower, indicating that flower tissue could provide a valuable source of information about these compounds. 

## 3. Experimental Section

### 3.1. Plant Materials

*I. dentata* var. *albiflora* was grown at the experimental farm of the National Institute of Horticultural and Herbal Science, Rural Development Administration (Eumseong, Korea) in 2015. Different organs (flowers, stems, leaves, and roots) were harvested from 15 plants in August 2015. All samples were immediately frozen in liquid nitrogen and then stored at −80 °C and/or freeze-dried for RNA isolation and/or high-performance liquid chromatography-ultraviolet (HPLC-UV) analysis. Five plants for each replicate were used for biological repeats. 

### 3.2. Total RNA Extraction and Quantitative Real Time-PCR (qRT-PCR)

Total RNA was extracted from the flowers, stems, leaves, and roots of *I. dentata* using an Easy BLUE Total RNA Kit (iNtRON, Seongnam, Korea). RNA quantity was measured on a NanoVue Plus spectrophotometer (GE Healthcare Life Sciences, Pittsburgh, PA, USA), and its quality was confirmed by electrophoresis of 1 μg of the RNA on a 1.2% formaldehyde RNA agarose gel. Then, 1 μg of total RNA was reverse-transcribed using a ReverTra Ace-α Kit (Toyobo, Osaka, Japan) and oligo (dT)_20_ primers. The protocol for the reverse transcriptase polymerase chain reaction (RT-PCR) was as follows: 11 μL of RNase-free water was mixed with 1 μg of total RNA (1 μL), 2 μL of 10× buffer, 1 μL of 10 mM of each dNTP, 2 μL of 10 μM oligo dT primer, 1 μL of 40 μ/μL RNase inhibitor, and 1 μL of 4 u/μL reverse transcriptase to a final volume of 20 μL. The mixture was reamplified for 20 min at 42 °C and heated for 5 min at 99 °C. The cDNA was diluted 20-fold for qRT-PCR. QRT-PCR was performed in a 20-μL reaction volume containing 0.5 μM primers and 2× Real-Time PCR Smart mix (SolGent, Daejeon, Korea). The qRT-PCR program was as follows: initial denaturation at 95 °C for 15 min followed by 40 cycles of denaturation at 95 °C for 20 s, annealing at 55 °C for 40 s, and extension at 72 °C for 20 s. The qRT-PCR results were obtained using the mean of three replicates. The *actin* gene (GenBank accession No. KY419710) was used for normalizing the real-time PCR data that has been used in numerous studies previously [37,38]. We used another reference gene 18S also which was not producing stable and reliable results, thus we adopted actin gene to pursue the experiments. Copy numbers of mRNA products were calculated using absorbance values. The mRNA products were sequentially diluted to prepare standard mRNA solutions, and were subjected to reverse transcription. Standard mRNA solutions were referred to as standard cDNA solutions and the PCR efficacy values were calculated by a free online tool (http://cels.uri.edu/gsc/cndna.html). In the qRT-PCR assay, several doses of standard cDNAs (1 × 10^7^ to 1 × 10^3^ copies for all cDNAs) were applied in triplicate in each run PCR. The data was analyzed with the Bio-Rad PCR machine inbuilt CFX Manager 2.0 software and the obtained values were plotted on log2 graph. 

### 3.3. Sequence Analysis

The deduced amino acid sequences of each gene were aligned using BioEdit (Biological sequence alignment editor). MWs and p*I* values were calculated using the Compute p*I*/Mw tool (http://ca.expasy.org/tools/pi_tool.html). Gene-specific primers were designed using an online program (https://www.genscript.com/ssl-bin/app/primer) (Table 1).

### 3.4. Extraction and Analysis of Phenylpropanoids from I. dentata var. albiflora

HPLC analysis of phenylpropanoids was carried using a previously published protocol with minor modifications [15]. Samples were first freeze-dried and ground into a fine powder. The same powder used for RNA extraction was used for phenylpropanoid analysis. Each sample (100 mg dry weight (DW)) was then extracted using 5 mL of 80% methanol (MeOH) containing 10% acetic acid (0.1% *v*/*v*). This step was followed by vortexing for 5 min at room temperature, and then the sample was extracted for 1 h at 37 °C, with 1 min of vortexing every 20 min. After centrifugation at 1000× *g* for 5 min, the supernatant was filtered and used for analysis. External standards were purchased from Extrasynthese (Genay, France). MeOH and acetic acid were purchased from Wako Pure Chemical Industries (Osaka, Japan) and Junsei Chemical Co., Ltd. (Kyoto, Japan), respectively. The extract was filtered through a 0.45-μm poly-filter and diluted two-fold with MeOH prior to HPLC analysis. Samples were analyzed using a Futecs model NS-4000 HPLC apparatus (Daejeon, Korea) with an UV−Vis detector and autosampler. The analysis was monitored at 280 nm and performed using a C18 column (250 × 4.6 mm, 5 μm; RStech; Daejeon, Korea). The mobile phase consisted of a mixture of (A) MeOH/water/acetic acid (5:92.5:2.5, *v*/*v*/*v*) and (B) MeOH/water/acetic acid (95:2.5:2.5, *v*/*v*/*v*), as described previously [21]. The initial mobile phase composition was 0% solvent B, followed by a linear gradient from 0 to 80% of solvent B in 48 min, then holding at 0% solvent B for an additional 10 min, and the column was maintained at 30 °C. The flow rate was set at 1.0 mL/min, and the injection volume was 20 μL. Quantification of the different compounds was based on peak areas and calculated as equivalents of representative standard compounds. All contents are expressed as micrograms per gram DW.

### 3.5. Statistical Analysis

All statistical analyses were performed using the statistical analysis software SPSS 17.0 (SPSS Inc., Chicago, IL, USA, 2009). All data are given as the mean and standard deviation of triplicate experiments. The significant differences among means were determined by Duncan’s Multiple Range Test. 

## 4. Conclusions

In this study, *IdPALs*, *IdC4H*, *Id4CLs*, *IdCHS*, and *IdDFR* were cloned and characterized from *I. dentata* using NGS. We investigated the transcript levels of the five genes using qRT-PCR, and analyzed the accumulation of phenylpropanoids in different organs of *I. dentata* using HPLC. The transcript levels of *IdPAL1*, *IdC4H*, *Id4CL1*, *IdCHS*, and *IdDFR* were highest in the leaf. In addition, catechin, chlorogenic acid, ferulic acid, and quercetin contents were highest in the leaf. We suggest that the expression of *IdPAL1*, *IdC4H*, *Id4CL1*, *IdCHS*, and *IdDFR* is associated with phenylpropanoid biosynthesis. Our results will provide baseline information for elucidating the mechanism of phenylpropanoid biosynthesis in the different organs of *I. dentata*.

## Figures and Tables

**Table 1:** Primers used in this study.

Primer Name	Sequence (5′ → 3′)	GenBank Access No.	Size (bp)
*IdPAL1(F)*	AGCTCCGGCAAGTTCTGGTC	KU724084	100
*IdPAL1(R)*	TCGTCTTCGAATGTGGCGAT		
*IdPAL2(F)*	CGCCTGGAGAGGAGTTCGAT	KU724085	103
*IdPAL2(R)*	GGAAGTGGAGTCCCATCCCA		
*IdC4H(F)*	ACCCAGGGCGTTGAGTTCGG	MF034078	114
*IdC4H(R)*	TCGCATCTTCCGCCAGTGCT		
*Id4CL1(F)*	ACAATCGCGAACAACGAGGA	KU724086	129
*Id4CL1(R)*	CTTCGCATTCGGGAACTTCA		
*Id4CL2(F)*	CGTCGCACAACAAGTCGATG	KU724087	103
*Id4CL2(R)*	CATCAGCACCACCGAAGCTC		
*IdCHS(F)*	CCAAGCTCCTCGGTCTTCGT	KU724088	104
*IdCHS(R)*	CTCAGCAAGGTCCTTCGCAA		
*IdDFR(F)*	ATTGGCGGAAAGGGCAGCAT	MF034079	113
*IdDFR(R)*	GGCTTGGAGGGAATGAGGGAGT		
*IdActin(F)*	CAATGGAACCGGAATGGTCA	KY419710	100
*IdActin(R)*	CATTACGCCCGTGTGTCGAG		

## Supplementary Materials

Supplementary materials are available online.

## References

[1] Ahn E.M., Bang M.H., Song M.C., Park M.H., Kim H.Y., Kwon B.M., Baek N.I. (2006). Cytotoxic and ACAT-inhibitory sesquiterpene lactones from the root of *Ixeris dentata* forma *albiflora*. Arch. Pharm. Res..

[2] Manach C., Scalbert A., Morand C., Remesy C., Jimenez L. (2004). Polyphenols: Food sources and bioavailability. Am. J. Clin. Nutr..

[3] Scalbert A., Johnson I.T., Saltmarsh M. (2005). Polyphenols: Antioxidants and beyond. Am. J. Clin. Nutr..

[4] Farah A., Donangelo C. (2006). Phenolic compounds in coffee. Braz. J. Plant Physiol..

[5] Winkel-Shirley B. (2001). Flavonoid biosynthesis. A colorful model for genetics, biochemistry, cell biology, and biotechnology. Plant Physiol..

[6] Vrhovsek U., Masuero D., Gasperotti M., Franceschi P., Caputi L., Viola R., Mattivi F. (2012). A versatile targeted metabolomics method for the rapid quantification of multiple classes of phenolics in fruits and beverages. J. Agric. Food Chem..

[7] Rice-Evans C.A., Miller N.J., Paganga G. (1996). Structure-antioxidant activity relationships of flavonoids and phenolic acids. Free Radic. Biol. Med..

[8] Rozema J., Björn L.O., Bornman J.F., Gaberscik A., Häder D.P.L., Trost T., Germ M., Klisch M., Gröniger A., Sinha R.P. (2002). The role of UV-B radiation in aquatic and terrestrial ecosystems—An experimental and functional analysis of the evolution of UV-absorbing compounds. J. Photochem. Photobiol. B.

[9] Cheynier V. (2012). Phenolic compounds: From plants to foods. Phytochem. Rev..

[10] Zhong C., Hu D., Hou L.B., Song L.Y., Zhang Y.J., Xie Y., Tian L.W. (2017). Phenolic compounds from the rhizomes of *Smilax china* L. and their anti-inflammatory activity. Molecules.

[11] Martens S., Teeri T., Forkmann G. (2002). Heterologous expression of dihydroflavonol 4-reductases from various plants. FEBS Lett..

[12] Strack D., Wray V. (1989). Methods in Plant Biochemistry.

[13] Li X., Park N.I., Xu H., Woo S.-H., Park C.H., Park S.U. (2010). Differential expression of flavonoid biosynthesis genes and accumulation of phenolic compounds in common buckwheat (*Fagopyrum esculentum*). J. Agric. Food Chem..

[14] Zhao S., Park C.H., Li X., Kim Y.B., Yang J., Sung G.B., Park N.I., Kim S., Park S.U. (2015). Accumulation of rutin and betulinic acid and expression of phenylpropanoid and triterpenoid biosynthetic genes in Mulberry (*Morus alba* L.). J. Agric. Food Chem..

[15] Park N.I., Xu H., Li X., Jang I.H., Park S.U. (2011). Anthocyanin accumulation and expression of anthocyanin biosynthetic genes in radish (*Raphanus sativus*). J. Agric. Food Chem..

[16] Kim Y.J., Kim Y.B., Li X., Choi S.R., Park S., Park J.S., Lim Y.P., Park S.U. (2015). Accumulation of phenylpropanoids by white, blue, and red light irradiation and their organ-specific distribution in Chinese cabbage (*Brassica rapa* ssp *pekinensis*). J. Agric. Food Chem..

[17] Schuster B., Retey J. (1994). Serine-202 is the putative precursor of the active site dehydroalanine of phenylalanine ammonia lyase. Site-directed mutagenesis studies on the enzyme from parsley (*Petroselinum crispum* L.). FEBS Lett..

[18] Stuible H.P., Kombrink E. (2001). Identification of the substrate specificity-conferring amino acid residues of 4-coumarate: Coenzyme A ligase allows the rational design of mutant enzymes with new catalytic properties. J. Biol. Chem..

[19] Schneider K., Hovel K., Witzel K., Hamberger B., Schomburg D., Kombrink E., Stuible H.P. (2003). The substrate specificity-determining amino acid code of 4-coumarate: CoA ligase. Proc. Natl. Acad. Sci. USA.

[20] Zhou B., Wang Y., Zhan Y., Li Y., Kawabata S. (2013). Chalcone synthase family genes have redundant roles in anthocyanin biosynthesis and in response to blue/UV-A light in turnip (*Brassica rapa*; Brassicaceae). Am. J. Bot..

[21] Li X., Kim J.K., Park S.-Y., Zhao S., Kim Y.B., Lee S., Park S.U. (2014). Comparative analysis of flavonoids and polar metabolite profiling of tanno-original and tanno-high rutin buckwheat. J. Agric. Food Chem..

[22] Tuan P.A., Park N.I., Li X., Xu H., Kim H.M., Park S.U. (2010). Molecular cloning and characterization of phenylalanine ammonia-lyase and cinnamate 4-hydroxylase in the phenylpropanoid biosynthesis pathway in garlic (*Allium sativum*). J. Agric. Food Chem..

[23] Xu H., Park N.I., Li X., Kim Y.K., Lee S.Y., Park S.U. (2010). Molecular cloning and characterization of phenylalanine ammonia-lyase, cinnamate 4-hydroxylase and genes involved in flavone biosynthesis in *Scutellaria baicalensis*. Bioresour. Technol..

[24] Kim Y.B., Shin Y.J., Tuan P.A., Li X., Park Y., Park N.I., Park S.U. (2014). Molecular cloning and characterization of genes involved in rosmarinic acid biosynthesis from *Prunella vulgaris*. Biol. Pharm. Bull..

[25] Zhao S., Li X., Cho D.H., Arasu M.V., Al-Dhabi N.A., Park S.U. (2014). Accumulation of kaempferitrin and expression of phenylpropanoid biosynthetic genes in kenaf (*Hibiscus cannabinus*). Molecules.

[26] Zhao S., Tuan P.A., Li X., Kim Y.B., Kim H., Park C.G., Yang J., Li C.H., Park S.U. (2013). Identification of phenylpropanoid biosynthetic genes and phenylpropanoid accumulation by transcriptome analysis of *Lycium chinense*. BMC Genom..

[27] Richet N., Tozo K., Afif D., Banvoy J., Legay S., Dizengremel P., Cabane M. (2012). The response to daylight or continuous ozone of phenylpropanoid and lignin biosynthesis pathways in poplar differs between leaves and wood. Planta.

[28] Park W.T., Arasu M.V., Al-Dhabi N.A., Yeo S.K., Jeon J., Park J.S., Lee S.Y., Park S.U. (2016). Yeast extract and silver nitrate induce the expression of phenylpropanoid biosynthetic genes and induce the accumulation of rosmarinic acid in *Agastache rugosa* cell culture. Molecules.

[29] Kim Y.B., Thwe A.A., Kim Y., Li X., Cho J.W., Park P.B., Arasu M.V., Al-Dhabi N.A., Kim S.J., Suzuki T. (2014). Transcripts of anthocyanidin reductase and leucoanthocyanidin reductase and measurement of catechin and epicatechin in tartary buckwheat. Sci. World J..

[30] Kalinova J., Vrchotova N. (2009). Level of catechin, myricetin, quercetin and isoquercitrin in buckwheat (*Fagopyrum esculentum Moench*), changes of their levels during vegetation and their effect on the growth of selected weeds. J. Agric. Food Chem..

[31] Li X., Kim Y.B., Kim Y., Zhao S., Kim H.H., Chung E., Lee J.H., Park S.U. (2013). Differential stress-response expression of two flavonol synthase genes and accumulation of flavonols in tartary buckwheat. J. Plant Physiol..

[32] Tuan P.A., Park W.T., Xu H., Park N.I., Park S.U. (2012). Accumulation of tilianin and rosmarinic acid and expression of phenylpropanoid biosynthetic genes in *Agastache rugosa*. J. Agric. Food Chem..

[33] Rani A., Singh K., Sood P., Kumar S., Ahuja P. (2009). *p*-Coumarate: CoA ligase as a key gene in the yield of catechins in tea [*Camellia sinensis*, L., O. KUNTZE]. Funct. Integr. Genom..

[34] Singh K., Kumar S., Rani A., Gulati A., Ahuja P. (2009). Phenylalanine ammonia-lyase (PAL) and cinnamate 4-hydroxylase (C4H) and catechins (flavan-3-ols) accumulation in tea. Funct. Integr. Genom..

[35] Xu F., Cai R., Cheng S., Du H., Wang Y., Cheng S. (2008). Molecular cloning, characterization and expression of phenylalanine ammonia-lyase gene from *Ginkgo biloba*. Afr. J. Biotechnol..

[36] Park J.H., Park N.I., Xu H., Park S.U. (2010). Cloning and characterization of phenylalanine ammonia-lyase and cinnamate 4-hydroxylase and pyranocoumarin biosynthesis in *Angelica gigas*. J. Nat. Prod..

[37] Tähtiharju S., Rijpkema A.S., Vetterli A., Albert V.A., Teeri T.H., Elomaa P. (2012). Evolution and diversification of the CYC/TB1 gene family in Asteraceae—A comparative study in Gerbera (Mutisieae) and sunflower (Heliantheae). Mol. Biol. Evol..

[38] Qi S., Yang L., Wen X., Hong Y., Song X., Zhang M., Dai S. (2016). Reference gene selection for RT-qPCR analysis of flower development in Chrysanthemum morifolium and Chrysanthemum lavandulifolium. Front. Plant Sci..

