# Supplementary material for: Expression of Genes Related to Phenylpropanoid Biosynthesis in Different Organs of Ixeris dentata var. albiflora
Source: Molecules. 2017 May 30;22(6):901. doi: 10.3390/molecules22060901 (PMC6152745; doi:10.3390/molecules22060901)
Supplement: Supplementary file 1 [file molecules-22-00901-s001.pdf]

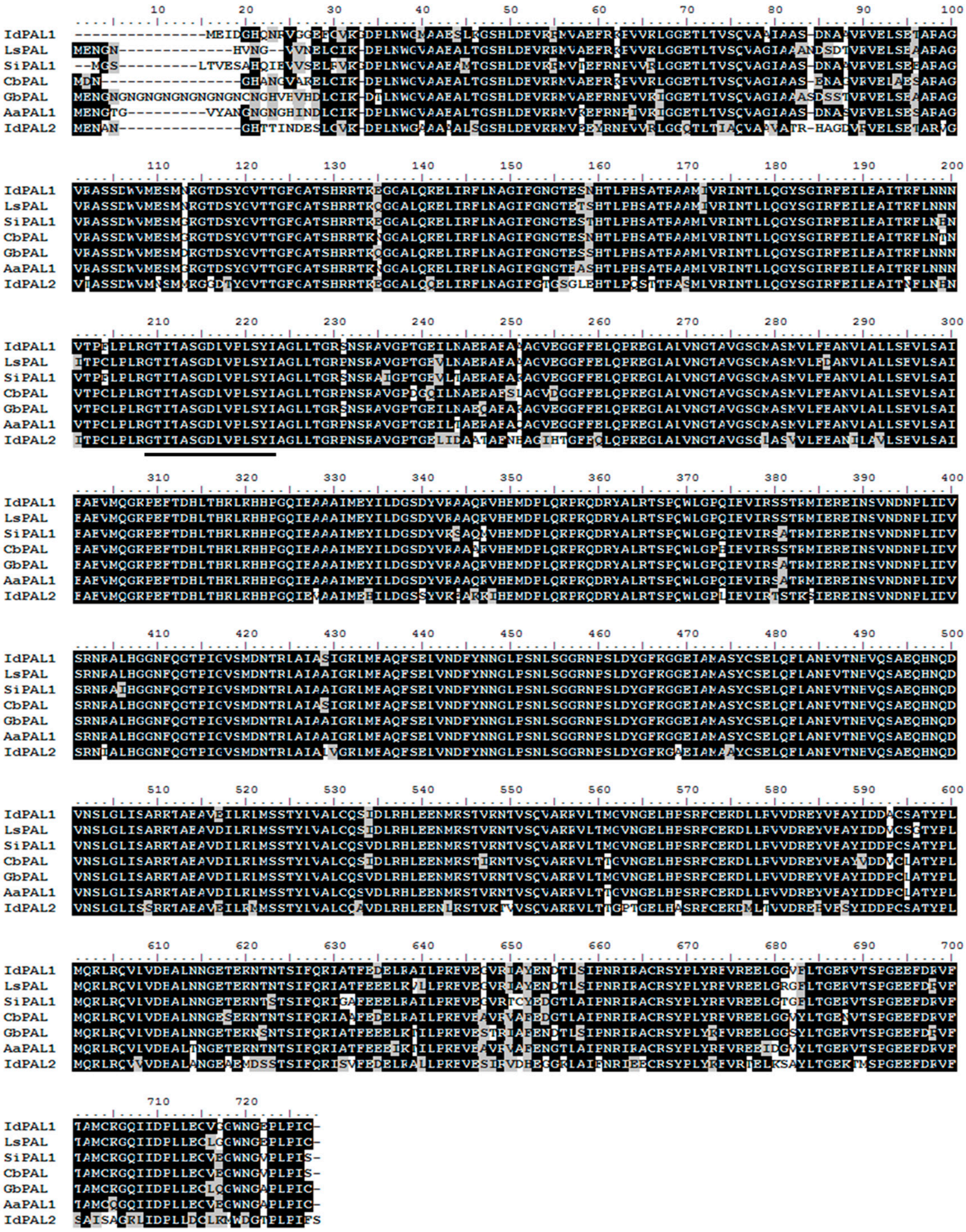

Figure S1. Multiple alignments of the amino acid sequences of IdPAL1 and IdPAL2 with those of other plants PAL. Identical residues are indicated by a black background and similar residues are shaded with a gray background. IdPAL1 (I. dentata, KU724084); LsPAL (Lactuca sativa, AAL55242); SiPAL1 (Saussurea involucrata, ALK02780); CbPAL (Chrysanthemum boreale, AGU91428); MoPAL (Gynura bicolor, BAJ17655); AaPAL1 (Artemisia annua, AKP55356); IdPAL2 (I. dentata, KU724085). The black bold underline indicates the conserved PAL motif.

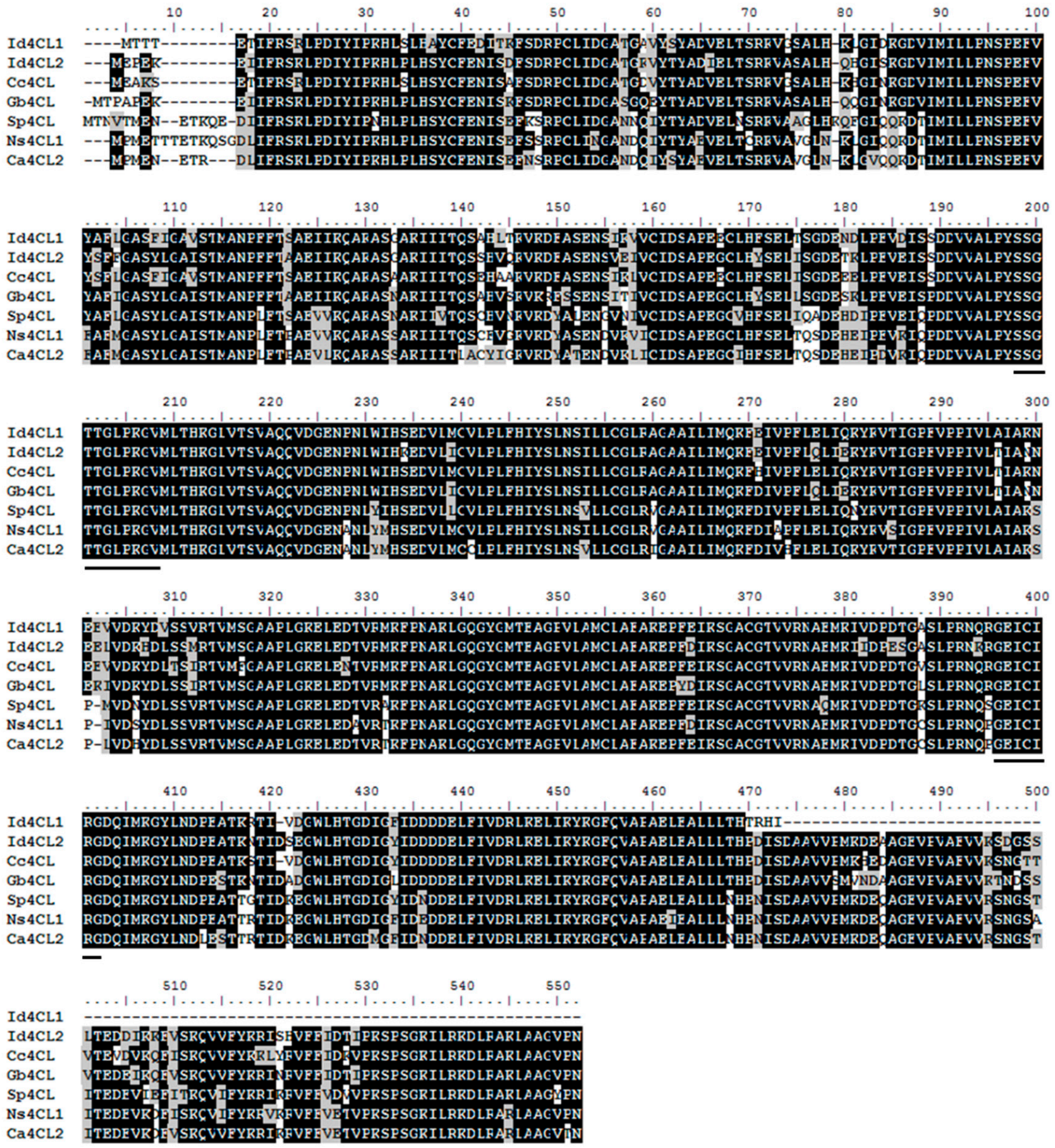

Figure S2. Multiple alignments of the amino acid sequences of Id4CL1 and Id4CL2 with those of other plants 4CL. Identical residues are indicated by a black background and similar residues are shaded with a gray background. Id4CL1(I. dentata, KU724086); Id4CL2(I. dentata, KU724087) Cc4CL (Cynara cardunculus, AFL93685); Gb4CL (Gynura bicolor, BAJ17664); Sp4CL (Solanum pennellii, XP_015080149); Ib4CL (Nicotiana sylvestris, XP_009798863); Ca4CL (Capsicum annuum, ACF17632). The black bold underline indicates the conserved 4CL motif.

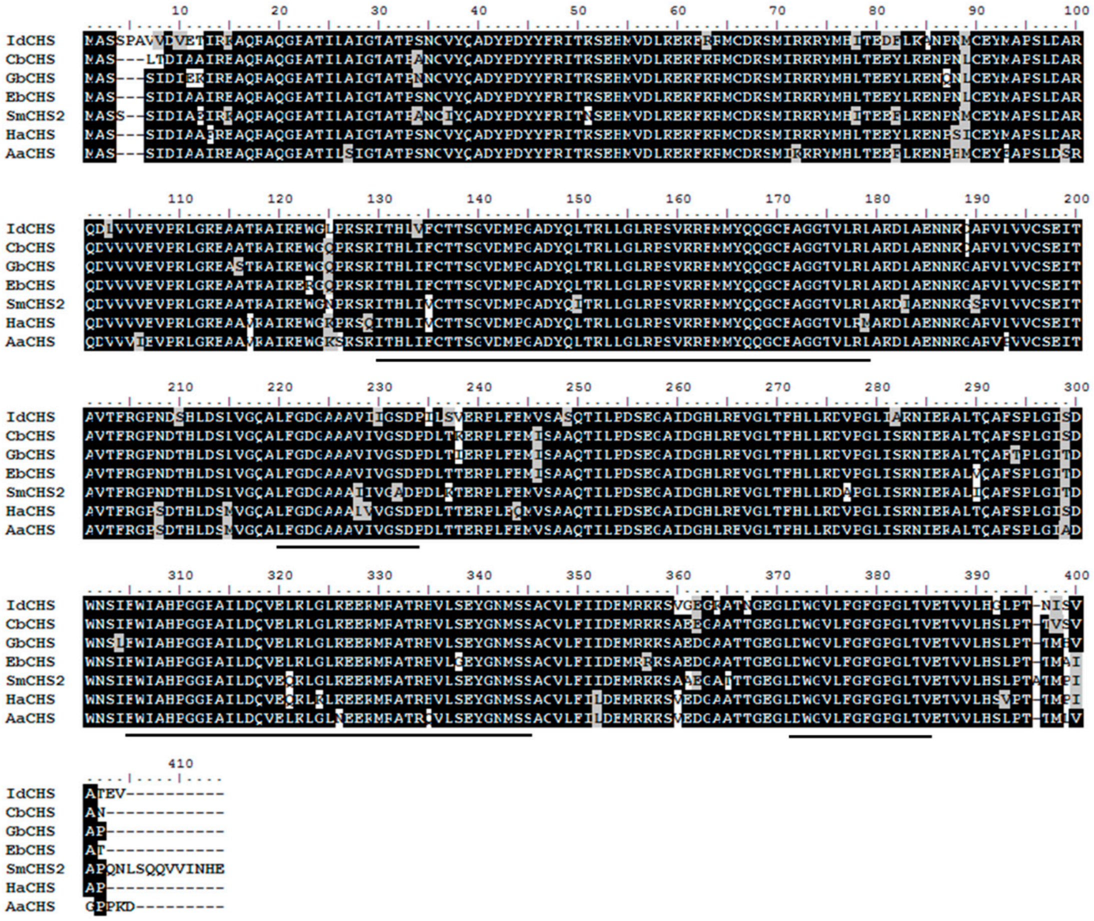

Figure S3. Multiple alignments of the amino acid sequence of IdCHS with those of other plants CHS. Identical residues are indicated by a black background and similar residues are shaded with a gray background. IdCHS (I. dentata, KU724088); CbCHS (Chrysanthemum boreale, AGU91424); GbCHS (Gynura bicolor, BAJ17656); EbCHS (Eschenbachia blinii, AHN85848); SmCHS (Silybum marianum, AFK65634); HaCHS (Helianthus annuus, ALL34489); AaCHS (Ageratina adenophora, ACQ84148). The black bold underline indicates the conserved CHS motif.
